# Immune-associated pivotal biomarkers identification and competing endogenous RNA network construction in post-operative atrial fibrillation by comprehensive bioinformatics and machine learning strategies

**DOI:** 10.3389/fimmu.2022.974935

**Published:** 2022-10-20

**Authors:** Yufei Zhou, Qianyun Wu, Gehui Ni, Yulu Hong, Shengjue Xiao, Chunjiang Liu, Zongliang Yu

**Affiliations:** ^1^ Department of Cardiology, Zhongshan Hospital, Fudan University, Shanghai, China; ^2^ Department of Cardiology, The First People’s Hospital of Kunshan Affiliated to Jiangsu University, Suzhou, China; ^3^ Department of Cardiology, The First Affiliated Hospital of Nanjing Medical University, Nanjing, China; ^4^ Department of Computer Science and Technology, Central South University, Changsha, China; ^5^ Department of Cardiology, Zhongda Hospital, School of Medicine, Southeast University, Nanjing, China; ^6^ Department of General Surgery, Shaoxing People’s Hospital (Shaoxing Hospital of Zhejiang University), Shaoxing, China

**Keywords:** post-operative atrial fibrillation, machine learning, bioinformatics analysis, diagnosis, immune and inflammation, competing endogenous RNA network

## Abstract

**Background:**

Atrial fibrillation (AF) is the most common arrhythmia. Previous studies mainly focused on identifying potential diagnostic biomarkers and treatment strategies for AF, while few studies concentrated on post-operative AF (POAF), particularly using bioinformatics analysis and machine learning algorithms. Therefore, our study aimed to identify immune-associated genes and provide the competing endogenous RNA (ceRNA) network for POAF.

**Methods:**

Three GSE datasets were downloaded from the GEO database, and we used a variety of bioinformatics strategies and machine learning algorithms to discover candidate hub genes. These techniques included identifying differentially expressed genes (DEGs) and circRNAs (DECs), building protein-protein interaction networks, selecting common genes, and filtering candidate hub genes via three machine learning algorithms. To assess the diagnostic value, we then created the nomogram and receiver operating curve (ROC). MiRNAs targeting DEGs and DECs were predicted using five tools and the competing endogenous RNA (ceRNA) network was built. Moreover, we performed the immune cell infiltration analysis to better elucidate the regulation of immune cells in POAF.

**Results:**

We identified 234 DEGs (82 up-regulated and 152 down-regulated) of POAF *via* Limma, 75 node genes were visualized *via* PPI network, which were mainly enriched in immune regulation. 15 common genes were selected using three CytoHubba algorithms. Following machine learning selection, the nomogram was created based on the four candidate hub genes. The area under curve (AUC) of the nomogram and individual gene were all over 0.75, showing the ideal diagnostic value. The dysregulation of macrophages may be critical in POAF pathogenesis. A novel circ_0007738 was discovered in POAF and the ceRNA network was eventually built.

**Conclusion:**

We identified four immune-associated candidate hub genes (*C1QA, C1R, MET, and SDC4*) for POAF diagnosis through the creation of a nomogram and evaluation of its diagnostic value. The modulation of macrophages and the ceRNA network may represent further therapy methods.

## Introduction

Atrial fibrillation (AF) is one of the most prevalent cardiac arrhythmias. Previous studies focused primarily on identifying potential diagnostic biomarkers and treatment options for atrial fibrillation (AF) through large-scale clinical trials, fundamental research, and data mining (1), but few studies have concentrated on postoperative atrial fibrillation (POAF).

POAF is a common complication after cardiac surgery, affecting 20% to 40% of patients undergoing cardiac surgery and accounting for approximately one-third of secondary AF cases (2). The majority of POAF symptoms are mostly asymptomatic, paroxysmal and brief. The incidence peaks between two to four days after surgery and recurs frequently during the first post-operative week (3). It can cause adverse consequences, such as an increase in hospitalization days, expenses, and critical care unit time (4). Moreover, it is an independent predictor of postoperative mortality since it is related with a higher incidence of stroke and hemodynamic instability due to AF (5, 6). POAF is promoted when transient, postoperative triggers act on a vulnerable atrial substrate comprised of preoperative, perioperative, and postoperative remodeling processes. Several independent risk variables are related with POAF, including age, male gender, history of AF, and peri-operative predictors such as mitral valve surgery and intra-aortic balloon pump (7). These risk factors alter the sensitivity of the atrial substrate to the two primary AF mechanisms: ectopic firing owing to triggered activity and re-entry.

Despite the fact that various clinical researches have focused on identifying risk factors and predicting the prognosis of POAF, the immune-associated diagnostic biomarkers and underlying pathophysiology remain unknown. To the best of our knowledge, no previous studies analyzed POAF using public GEO datasets and machine learning algorithms.

Herein, after a series of bioinformatics analysis and machine learning algorithms, we identified four diagnostic biomarkers and created the nomogram for POAF prediction based on the receiver operating characteristic curve evaluation (ROC). Furthermore, we investigated the immune cell infiltration between POAF and sinus rhythm (SR). In addition, the circ/miR/mRNA network of POAF was built. In general, our study offered a systematic analysis of POAF, including identification of diagnostic markers, building of competing endogenous RNA (ceRNA) network, and examination of immune pathway components.

## Methods

### Microarray data

**Figure 1:**
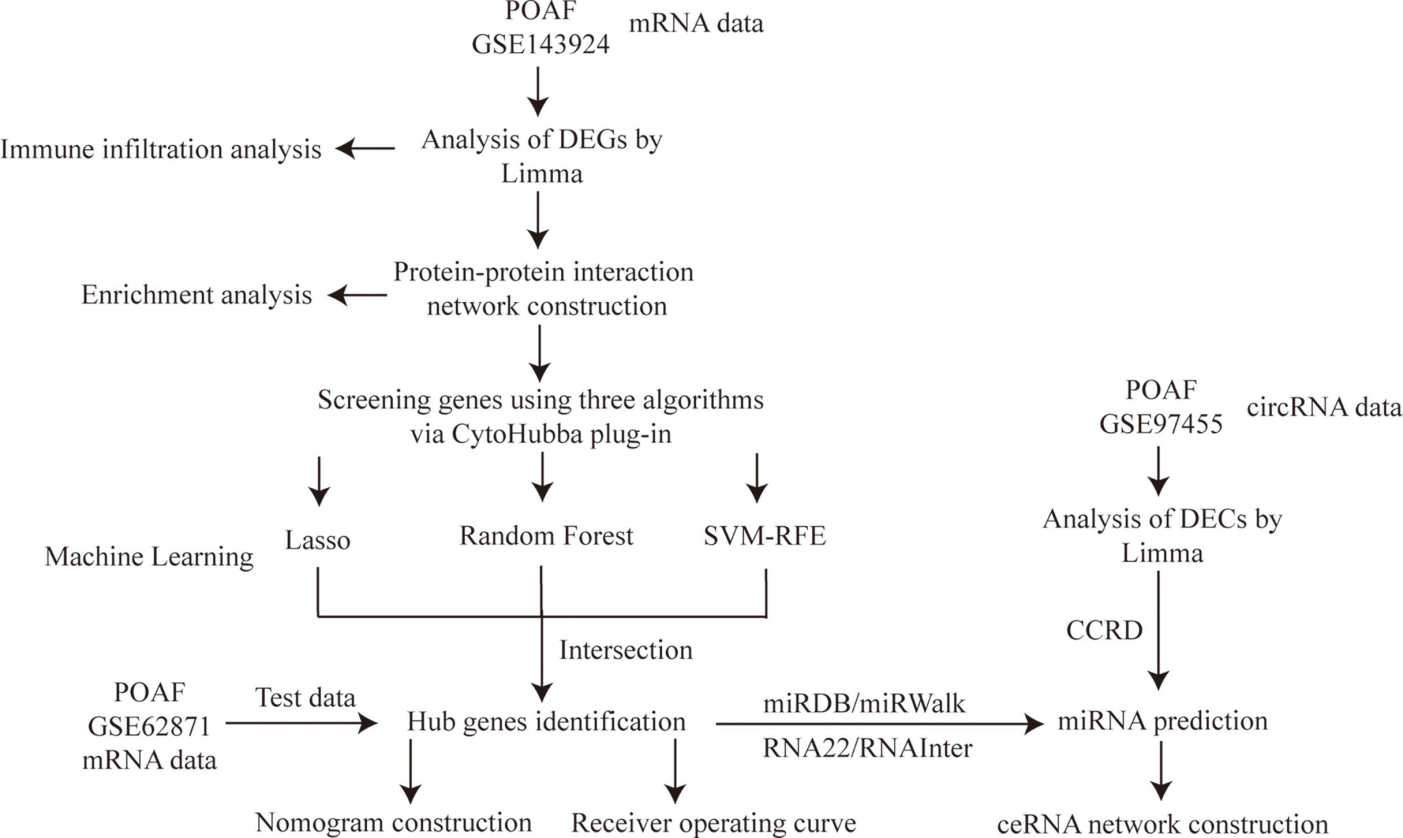
Study flowchart. POAF, post-operative atrial fibrillation; DEGs, differentially expressed genes; Limma, linear models for microarray data; Lasso, least absolute shrinkage and selection operator; SVM-RFE, support vector machine recursive feature elimination; DECs, differentially expressed circRNAs; CCRD, cancer-specific circRNA database; ceRNA, competing endogenous RNA.

The study flowchart is shown in **Figure 1**. (post operative atrial fibrillation) AND “Homo sapiens”[porgn:_ txid9606] was the search strategy for selecting appropriate datasets. Two gene expression datasets (GSE143924, GSE62871) and one non-coding RNA dataset (GSE97455) were downloaded from the NCBI Gene Expression Omnibus (GEO; https://www.ncbi.nlm.nih.gov/geo/) (8). GSE143924 was generated using Affymetrix Human Gene 2.0 ST Array and included epicardial adipose tissue from 30 participants (15 Sinus rhythm (SR) and 15 POAF). GSE62871 (9), generated from Agilent-039494 SurePrint G3 Human GE v2 8x60K, consisted of right atrial appendage from 16 participants (9 SR and 7 POAF). GSE97455 was generated using 074301 Arraystar Human CircRNA microarray V2 and contained plasma samples from 15 SR and 15 POAF.

### Differentially expressed genes and circRNAs identification

After downloading the raw datasets, background calibration, normalization, and log2 transformation were performed using affy in R software (version 4.1.3; https://www.r-project.org/) to preprocess the data. When multiple probes and different transcripts corresponded to the same common gene, the median value was calculated as its expression. The Bioconductor Limma (10) R package was used to identify DEGs and DECs. The criteria for identification of DEGs and DECs between SR and POAF were |Fold change| > 1.5 and P value < 0.05, |Fold change| > 2 and P value < 0.05, respectively.

### Protein-protein interaction network construction

The Search Tool for the Retrieval of Interacting Genes (String) database (11) (version 11.5; www.string-db.org), an online tool to identify the interaction among proteins, was used to construct PPI network. The minimum required interaction score was set as 0.40, the default value of String. Cytoscape (http://www.cytoscape.org) (12) software was applied to visualize the network and calculate the node genes subsequently. Three algorithms (Betweenness, Closeness, Degree) in CytoHubba, a plug-in of Cytoscape, were applied to identify top 20 genes. The intersection of 20 genes from the three algorithms were selected using Draw Venn diagram (http://bioinformatics.psb.ugent.be/webtools/Venn/) for further machine learning analysis.

### Functional enrichment analysis

Functional enrichment analysis include Gene Ontology (GO, http://geneontology.org) (13) and Kyoto Encyclopedia of Genes and Genomes (KEGG, https://www.kegg.jp/) (14). To explore the biological mechanisms of node genes regarding POAF, GO and KEGG enrichment analysis were performed using Sangerbox (http://www.sangerbox.com/home.html), a friendly and comprehensive clinical platform for bioinformatics analysis. The selection criteria included: number of genes enriched in the item > 2 and the adjusted P value < 0.05 to eliminate the bias caused by a large gene list input or enriched items containing a large number of genes.

### Machine learning

Herein, to minimize the risk of bias in potential diagnostic genes, three different machine learning algorithms were applied to identify candidate hub genes. Lasso (15) is a regression analysis approach using regularization to reduce the prediction error and was performed *via* “glmnet” R package. Random forest (16) is a popular classification and regression method in biological research, it can generate one decision tree forest and screen out critical genes through 10-fold cross validation method. Support vector machine recursive feature elimination (SVM-RFE) (17) is a supervised machine-learning algorithm with high accuracy to identify the most suitable characteristic genes. The intersection of genes from the three algorithms were selected for nomogram construction.

### Nomogram construction and receiver operating characteristic curve building

The ROC curve was initially established using SPSS Version 26.0 (IBM Corporation, Armonk, NY, USA) to visualize the diagnostic value of each individual gene with the calculation of area under curve (AUC) and 95% confidence interval (CI). The other POAF and SR validation cohort was applied to further validate its value. Then, the nomogram was constructed using “rms” R package to provide clinical perspective in POAF diagnosis. Each gene possesses the score based on its expression and after the accumulation of all genes, the total score can be used to predict POAF occurrence risk from the nomogram.

### MiRNAs targeting DEGs and DECs prediction

After a series systematical bioinformatics analysis and machine learning algorithms identification, hub genes for ceRNA network construction were selected. Therefore, to present a more precise ceRNA network, only DECs with |Fold change| > 4 were selected for ceRNA network construction. Four databases, including miRDB, miRWalk, RNA22, and RNAInter (18, 19), were used to predict miRNAs targeting DEGs. Meanwhile, miRNAs targeting DECs were predicted using CCRD (20).

### Circ-miR-mRNA network building

After the prediction of miRNAs targeting DEGs and DECs, only the intersection of miRNAs targeting DEGs from four databases were selected for ceRNA network construction. By the integration of the circRNA/miRNA pairs and the miRNA/mRNA pairs, a ceRNA regulation network was constructed. Nodes that cannot complete a circRNA-miRNA-mRNA axis were removed. Cytoscape was used to visualize the network.

### Immune cell infiltration

CIBERSORT is a computational method for distinguishing 22 human immune cell phenotypes (21). The “Cibersort” R package was applied to compute and depict the percentage of each immune cell type in various samples. To compare and visualize the proportion of 22 immune cells between POAF and SR, the “boxplot” R software was used. Using the “corrplot” R package, a correlation heatmap displaying the correlation of 22 types of infiltrating immune cells was generated (22)..

## Results

### DEGs identification *via* Limma

**Figure 2:**
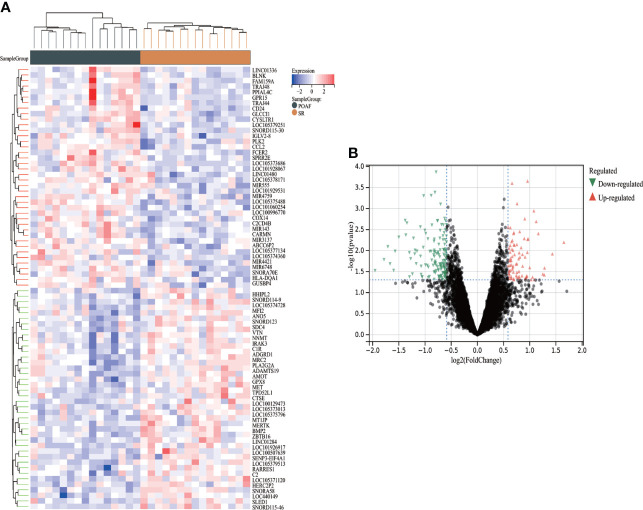
The heatmap and volcano plot of DEGs for POAF compared with SR in GSE143924. **(A)** Heatmap reveals top 25 up- and down-regulated DEGs for POAF patients compared with SR. Red and black represent up- and down- expression, respectively. **(B)** The volcano plot shows all DEGs for POAF compared with SR. Red and green triangles represent the significant DEGs following the filtration criteria. SR, sinus rhythm; others see **Figure 1**.

A total of 234 DEGs between POAF and SR in GSE143924 were identified using Limma package, of which 82 were up-regulated and 152 were down-regulated. The complete list of DEGs was provided in **Supplementary Table S1**. **Figure 2A, B** display the heatmap of the top 25 up- and down- regulated DEGs and the volcano plot for all DEGs.

### PPI network construction and common genes identification

**Figure 3:**
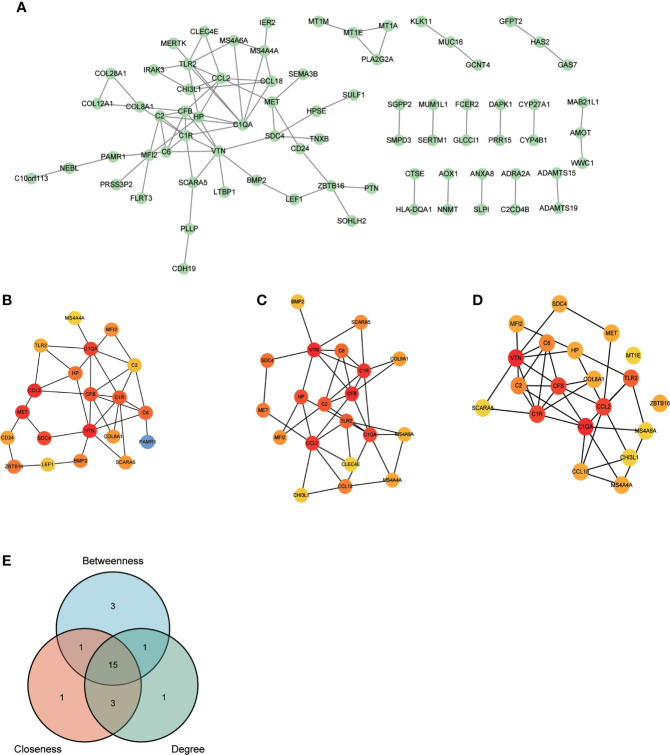
PPI network construction and common genes selection. **(A)** 75 POAF node genes were visualized from the PPI network. **(B-D)** Top 20 node genes were visualized using “Betweenness”, “Closeness”, and “Degree” algorithms *via* CytoHubba plug-in from Cytoscape, respectively. **(E)** The Venn diagram of top 20 node genes from three algorithms shows that 15 genes were identified as common genes for machine learning analysis after intersection. PPI, protein-protein interaction network; others see **Figure 1**.

After removing the isolated nodes and non-protein coding genes, 75 protein-coding node genes were identified in the PPI network and visualized *via* Cytoscape (**Figure 3**). The top 20 node genes from Betweenness, Closeness, and Degree algorithms using CytoHubba plug-in were visualized and presented in **Figure 3D**. The intersection of 20 genes from the aforementioned algorithms was depicted using Venn diagram and 15 genes were identified as common genes for candidate hub genes selection (**Figure 3**). The majority of them were intimately associated with immune and inflammation regulation. The detailed information of 15 common genes were listed as **Supplementary Table S2**.

### Functional enrichment analysis of node genes

**Figure 4:**
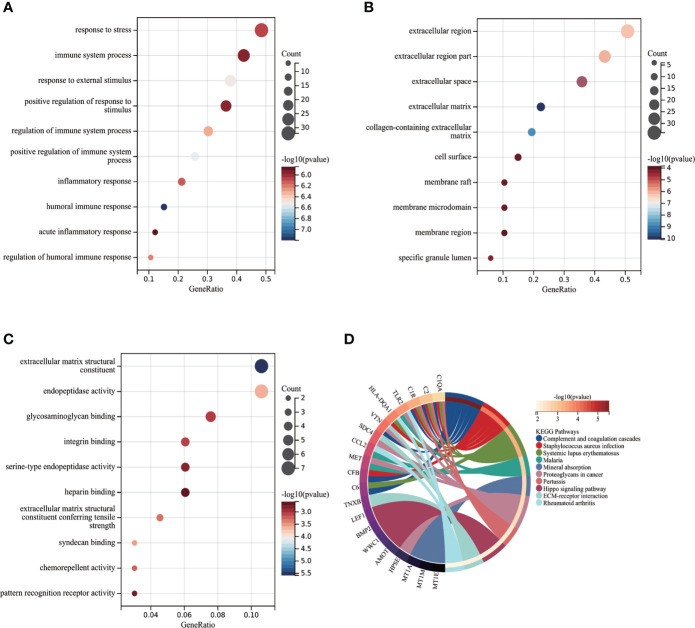
Functional enrichment analysis of POAF node genes. **(A-C)** Partial visualization of GO analysis for node genes from biological process, cellular component, and molecular function, respectively. X-axis represents gene ratio, Y-axis represents different ontologies, the circle color represents P-value and the circle size shows count number. **(D)** KEGG pathway analysis of node genes. The left side displays genes, while the right side depicts significant enriched pathways. The connection between genes and pathways refers to that genes were enriched in related pathway. POAF, post-operative atrial fibrillation; GO, gene ontology; KEGG, Kyoto Encyclopedia of Genes and Genomes.

Functional enrichment analysis was performed based on the 75 node genes. The GO analysis demonstrated that node genes were mainly enriched in “response to stress”, “immune system process”, “response to external stimulus” regarding BP (**Figure 4**); “extracellular region”, “extracellular region part”, “extracellular space” regarding CC (**Figure 4**); “extracellular matrix structural constituent”, “endopeptidase activity”, “glycosaminoglycan binding” regarding MF (**Figure 4**). The KEGG pathway enrichment analysis showed that complement and coagulation cascades play the critical role in POAF pathogenesis (**Figure 4**). The detailed results of the enrichment analysis were presented in **Supplementary Table S3**.

### Candidate hub genes selection *via* machine learning

SVM-RFE, random forest, and Lasso regression algorithms were applied to screen out candidate hub genes for POAF diagnosis.

**Figure 5:**
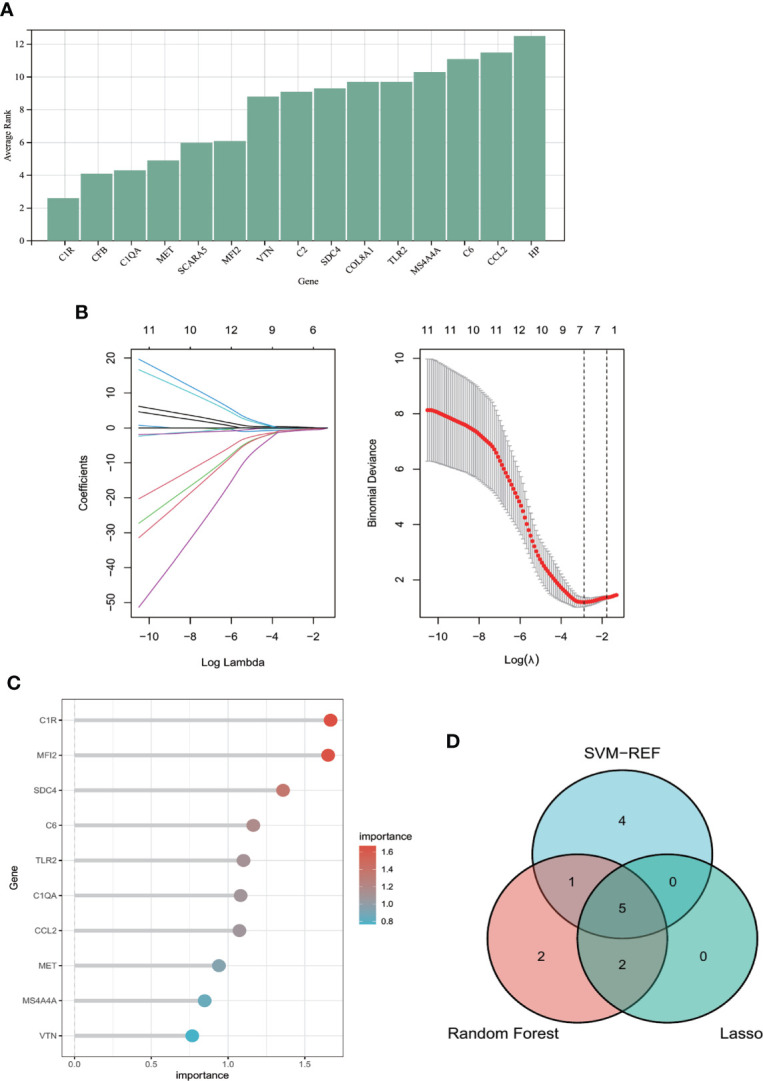
Machine learning algorithms for identifying candidate hub genes in diagnosing POAF. **(A)** Common genes were ranked based on the average rank using SVM-RFE algorithm after 100 folds. The lower the average rank, the greater the significance of the gene. **(B)** The number of genes (n=7) corresponding to the lowest point of the curve is the most suitable number for POAF diagnosis using Lasso algorithm. **(C)** The column reveals that genes were ranked with importance score using random forest algorithm. **(D)** The Venn diagram depicts the intersection of genes shared by three distinct methods. Five genes were chosen for nomogram development and diagnostic value assessment. POAF, post-operative atrial fibrillation; SVM-RFE, support vector machine-recursive feature elimination.

**Figure 5** shows the average rank of common genes after 100 folds using SVM-RFE algorithm. Genes with the smaller rank are more significant in POAF pathogenesis. The ten most important genes were chosen. **Figure 5** indicates that the LASSO regression algorithm identified seven potential candidate hub genes with the lowest binominal deviance. **Figure 5** reveals that genes were ranked based on the importance score calculation. The greater the score, the more significant the position.

The Venn diagram (**Figure 5**) showed that the intersection of the top ten common genes from SVM-RFE and random forest as well as seven genes from Lasso was five (*MET, C1QA, SDC4, C1R*, and *MFI2*), which were selected as the candidate hub genes for the further nomogram building and diagnostic value evaluation.

### Diagnostic value evaluation *via* ROC curve

**Figure 6:**
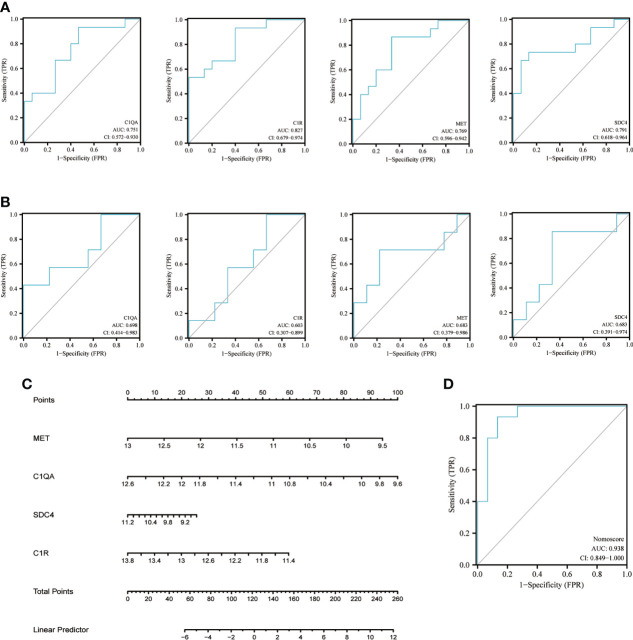
Nomogram construction and ROC evaluation. **(A)** The ROC curve of the individual candidate hub gene (*C1QA, C1R, MET, SDC4*) in nomogram construction. **(B)** The ROC curve of the same candidate hub gene derived from a different test dataset-GSE62871. **(C)** The nomogram was constructed based on the four candidate hub genes. **(D)** The ROC curve of the nomogram. ROC, receiver operating curve; C1QA, Complement C1qA Chain; C1R, Complement C1r; MET, MET Proto-Oncogene, Receptor Tyrosine Kinase; SDC4, Syndecan 4.

The diagnostic value of each candidate hub gene was assessed using ROC curves and revalidated using the other validation dataset GSE62871. *MFI2* was not observed in gene symbol after a succession of GSE62871 processing steps, which may be attributable to the use of different microarray platforms. Therefore, the ROC curve was determined using the remaining four candidate hub genes. **Figure 6** showed the AUC and 95% CI for each gene: *C1QA* (AUC: 0.751, CI 0.572-0.930), *C1R* (AUC: 0.827, CI 0.679-0.974), *MET* (AUC: 0.769, CI 0.596-0.942), *SDC4*(AUC: 0.791, CI 0.681-0.964). All the four candidate genes demonstrated optimal diagnostic value. Although the AUC for each gene was reduced after GSE62871 validation, this may be due to sample size constraints (**Figure 6**). Finally, the nomogram was built (**Figure 6**) and the AUC under nomogram was 0.938, showing the highest clinical diagnostic value (**Figure 6**).

### DECs identification *via* Limma

**Figure 7:**
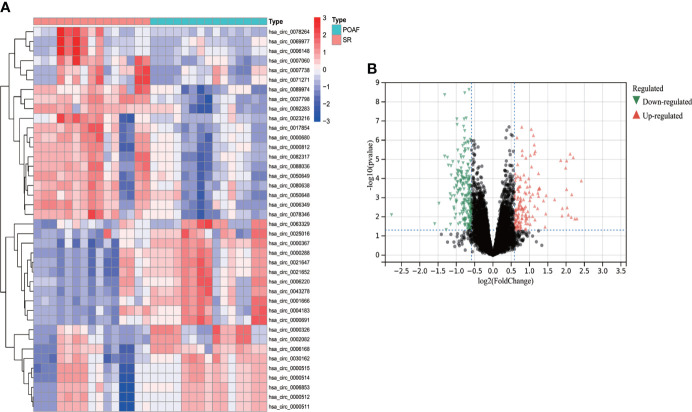
The heatmap and volcano plot of DECs for POAF compared with SR in GSE97455. **(A)** The heatmap displays top 20 up- and down-regulated DECs, Red and blue represent up- and down- expression. **(B)** The volcano plot showed all DECs for POAF. Red and green triangles refer to the significant DECs based on the selection criteria (Fold change > 2 and P value < 0.05). DELs, differentially expressed circRNAs; POAF, post-operative atrial fibrillation; SR, sinus rhythm.

68 DECs were identified using Limma method, of which 43 were up-regulated and 25 were down-regulated. The heatmap of top 20 up- and down-regulated DECs were shown in **Figure 7**, and the volcano plot for all DECs were displayed in **Figure 7**. The complete list of DECs was shown in **Supplementary Table S4**. 13 DECs (12 up-regulated and one down-regulated) were selected for ceRNA construction based on more rigorous DECs filtering criteria (|Fold change| > 4 and P value < 0.05).

### MiRNAs prediction and ceRNA network construction

Four candidate hub genes and 13 DECs were used to construct ceRNA network.

As circRNAs could bind miRNA competitively to regulate mRNA expression (23), only genes and DECs with similar expression trends were selected to create the ceRNA network. All four genes were downregulated in POAF; hence, only circ_0007738 was employed to form the ceRNA network.

**Figure 8:**
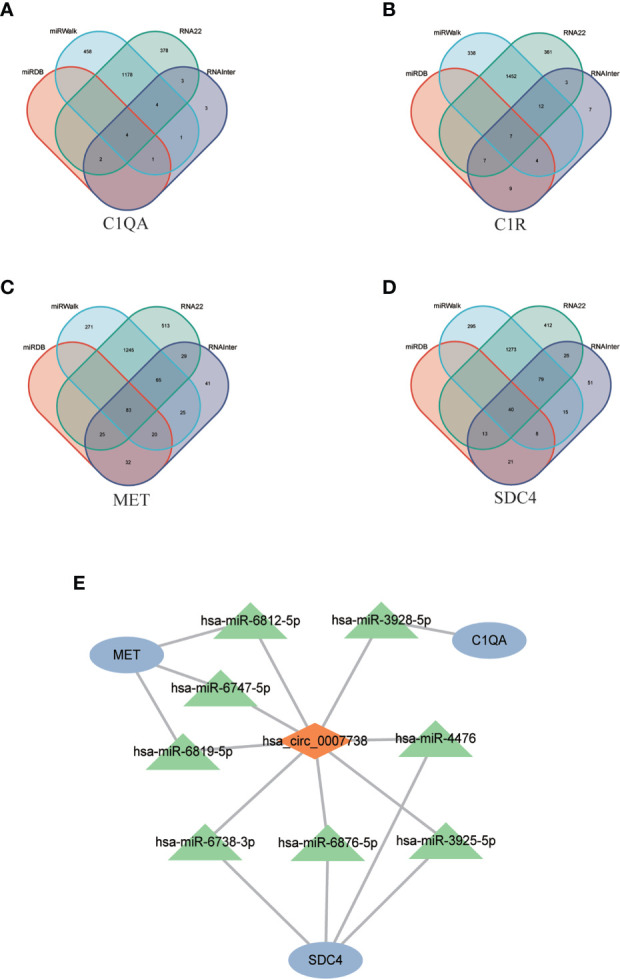
CeRNA network construction. **(A-D)** The Venn diagrams show the intersection of predicted miRNAs targeting mRNAs (*C1QA, C1R, MET, SDC4*) using four databases (miRDB, miRWalk, RNA22, RNAInter). **(E)** The constructed ceRNA network includes three mRNAs, eight predicted miRNAs, and one circRNAs. ceRNA, competing endogenous RNA; *C1QA*, Complement C1q A Chain; *C1R*, Complement C1r; *MET*, MET Proto-Oncogene, Receptor Tyrosine Kinase; *SDC4*, Syndecan 4.

Four databases were intersected to predict miRNAs that target mRNA, and the results indicated that four, seven, 83, and 40 miRNAs were predicted for *C1QA*, *C1R*, *MET*, and *SDC4*, respectively (**Figure 8D**). Eight miRNAs targeting circ_0007738 were identified. The detailed mRNA-miRNA and circRNA-miRNA pair information was provided in **Supplementary Table S5**. Finally, the ceRNA network was built, including three mRNAs, eight miRNAs and one circRNA (**Figure 8**).

### Immune cell infiltration analysis

Since the identified candidate hub genes were mainly enriched in immune regulation and the function description from gene cards were primarily about complement. We aimed to excavate immune cell infiltration in POAF.

**Figure 9:**
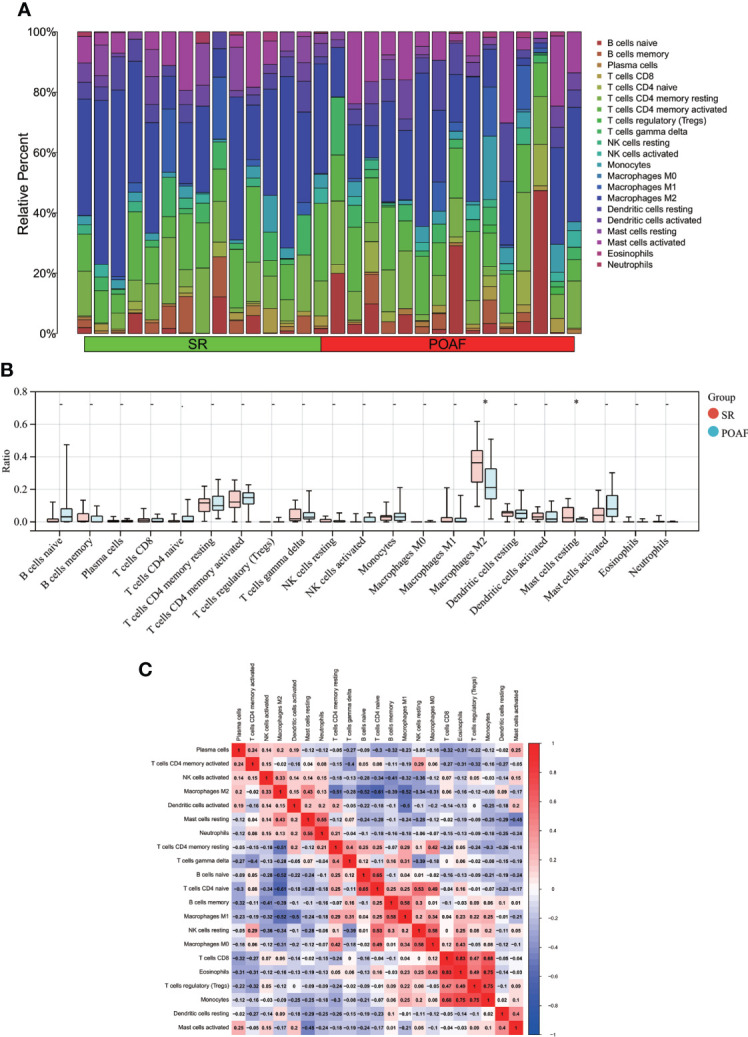
Immune cell infiltration between POAF and SR. **(A)** The proportion of 21 subtypes of immune cells in different samples regarding POAF and SR groups. **(B)** The barplot shows the comparison of 21 immune cell subtypes proportion between POAF and SR groups. Red and blue column represent SR and POAF group, respectively. *P < 0.05. **(C)** Correlation matrix of all 21 immune cell subtype compositions. The correlation coefficients are shown in the corresponding grids.

Following the use of the Cibersort algorithm, the percentage of 21 types of immune cells in each sample was displayed using a bar plot (Follicular help T cell was omitted because its proportion was equal to 0 in all samples) (**Figure 9**). The vioplot of the differential in immune cell infiltration revealed that POAF patients had a lower level of M2 macrophages and resting mast cells than SR (**Figure 9**). The correlation of 21 types of immune cells revealed that monocytes were positively related with Tregs (r = 0.75) and eosinophils (r = 0.75), whereas CD4 naïve T cells were negatively related with M2 macrophages (r = -0.61) (**Figure 9**). In summary, focusing on macrophage regulation could serve as potential approaches for POAF treatment.

## Discussion

The incidence of POAF is still high despite the progression of drug innovation in recent years. Our study discovered four biomarkers (*C1QA, C1R, MET*, and *SDC4*) and constructed a nomogram for clinical use in predicting the occurrence of POAF. The nomogram provides us with an intuitive and user-friendly interface to predict the risk of POAF. Based on the identified four biomarkers, we can collect blood samples from patients and test the expression of the four genes, each gene corresponds to a score according to its expression level and the accumulation of all scores refer to a total score that can be used to predict the incidence of POAF.

Despite the fact that the underlying mechanisms of POAF still remain elusive, the dysregulation of cardiac metabolism, epigenetic methylation, and the inflammation (24, 25) have been widely recognized. A series of clinic research in POAF prediction have found several classical indicators associated to cardiac metabolism. For instance, Yasushige et al. (26) revealed that lower expression of fatty acid binding protein 3 (*FABP3*), a regulator involved in fatty acids uptake (27), may be utilized to predict POAF regardless of age or atrial size. Furthermore, mitochondria are important in controlling cardiomyocyte energy metabolism. David et al. (9) proved that POAF was substantially linked with lower pre-operative mitochondrial respiration and higher sensitivity to calcium-induced mitochondrial permeability transition pore opening. DNA methylation is a type of chemical DNA modification that can change genetic activity without altering the DNA sequence (28). The Framingham Heart Study (29) clarified the association between DNA methylation and AF, and Matthew et al. (30) recently established DNA methylation biomarkers that can predict the incidence of POAF.

There is growing evidence that inflammation has a significant role in the incidence and recurrence of AF after cardiac surgery. Amar et al. (31) found that increments in white blood cell (WBC) were greater in patients with AF and coincided with the peak onset of POAF. A prospective study shows that an increase in postoperative WBC count predicts POAF development independently (32). In a pilot study of patients undergoing cardiac surgery, researchers identified peri-operative upregulation of the monocyte adhesion receptor, *CD11b*, and higher circulating monocyte as predictors for POAF (33). Similarly, the progression of POAF is closely correlated with changes in inflammatory cytokines. For example, Hak et al. (34). found a straightforward connection between interleukin *(IL)-2* serum levels and the development of POAF following coronary artery bypass grafting. It was also reported that the *IL-6* promoter gene variation modulates the inflammatory response to surgery and influences the development of POAF (35). Increased peripheral high-sensitive C reaction protein level has been identified to initiate intracardiac inflammatory state, cause atrium substrate abnormalities, and hence contribute to POAF (36). In summary, inflammation plays the critical role in POAF development, immunological response appears to have the capacity to predict POAF.

Herein, the four identified biomarkers in POAF were partially associated with inflammation and immune regulation. *C1QA* and *C1R* were both essential immune regulatory complement components. The complement system is a crucial component of innate and adaptive immunity for the identification and elimination of invading pathogen*s* (37). *C1Q* is the first recognition molecule regarding the classical pathway of the complement system. *C1Q* deficiency has been demonstrated to be vital in the incidence of various diseases. Ling et al. (38) revealed that *C1Q* could inhibit the response to self-antigens *via* altering the mitochondrial metabolism of CD8^+^ T lymphocytes in systemic lupus erythematosus, and *C1Q* deficiency may result in fatal immunopathology. While *C1QA* appears to be expressed differently in cancer. Azzato et al. (39) reported that overexpression of *C1QA* was strongly related with a favorable prognosis in oestrogen-receptor-negative cancers. Similarly, *C1R* expression has been reported to be highly associated with the pathogenesis of cardiovascular disease.

Patients with acute myocardial infarction showed coordinated proteomic signature changes in complement proteins (C1R) and immunological response, according to Cubedo et al. (40). The role of complement system in AF pathogenesis has been partially confirmed by Wen et al. (41). They found that the cascade response functions in the complement system were markedly diminished in AF patients, based on the downregulation of the mRNA expression of complement system genes. Our data showed that *C1QA* and *C1R* expression was lower in POAF than in SR, which is similar as AF, indicating that the innate and adaptive immune responses were impaired following cardiac surgery, resulting in POAF.

*MET* is a proto-oncogene and has been found to be critical in tumor regulation. Paolo (42) summarized that it could initiate and sustain neoplastic transformation when genetically altered. *MET* has been identified as a possible therapeutic target in the treatment of cancer. For instance, Alex et al. (43) found that in some genomic subsets of lung cancer, MET activation acted as both a primary and a secondary oncogenic driver of acquired resistance to targeted therapy. Also, it is crucial to concentrate on MET regulation in cardiac repair. *MET* deficiency in cardiomyocyte resulted in a reduction in anti-oxidant defense capacity. Meanwhile, the activation of *MET* after hypoxia-induced damage could activate the PI3K/AKT pathway and increase the activity of the MAPK cascades to exert an anti-apoptotic function (44). The downregulation of *MET* in POAF patients suggests that the anti-apoptotic function was weakened during cardiac surgery, resulting in the formation of POAF.

*SDC4* is a key membrane-associated endogenous receptor. Studies were primarily focused on human tumorigenesis and development due to its widely regulation of cytoskeleton, cell adhesion, and cell migration (45). Chen et al. (46) revealed that silencing *SDC4* promotes human papillary thyroid cancer cell death and inhibits epithelial mesenchymal transition *via* Wnt/β-Catenin pathway. Meanwhile, *SDC4* is important in regulating the formation of extracellular matrix, which provides the novel targets for cardiac fibrosis and the subsequent pathological remodeling induced by various stimuli. For instance, Kate et al. (47) discovered that *SDC4* can protect the heart from the profibrotic effects of thrombin-cleaved osteopontin. Even though there isn’t any concrete proof linking *SDC4* to POAF, Wu et al. (48) have explored the function of *SDC4* in AF. They discovered that individuals with valvular AF had downregulated *SDC4* expression, and that *SDC4* could control AF etiology by regulating oxidative stress and inflammatory responses. Our research revealed that *SDC4* was down-regulated in POAF patients, which is similar to AF, and this finding could suggest that extracellular matrix synthesis was impaired after cardiac surgery.

Due to the strong relationship between the four genes and immune regulation, our study also analyzed the immune infiltration of POAF. M2 and resting macrophages were down-regulated in POAF compared to SR, while the remaining 19 types of immune cells displayed the same trend. Previous research on the cross-talk between macrophages and atrial myocytes in AF found that the increased macrophages in the atrium were predominantly M1 pro-inflammatory macrophages, which is not consistent with our findings (49). We speculated that this may be attributable to the distinct immunological patterns of POAF and AF. To clarify the mechanism, additional basic research is required. In addition, we identified a circRNA and presented the POAF ceRNA network. The expression of circ_0007738 was reduced by more than fourfold in POAF. However, no articles pertaining to circ_0007738 have yet been discovered, requiring further investigation.

## Limitation and expectation

Our study had several limitations. First, there are only two mRNA datasets and one circRNA dataset available for POAF in GEO. These datasets were produced utilizing several platforms, and the variety of sample types resulted in bias that needed to be verified in our clinical samples. Second, given the various types of cardiac surgeries, it is uncertain whether the detected DEGs had the ideal diagnostic value for POAF following various cardiac surgeries. Third, additional clinical variables should be taken into account considering the limited clinical information provided by the datasets. To clarify POAF more clearly, we can add more clinical details to the nomogram. Finally, it is essential to investigate the underlying mechanism linking the detected DEGs, DECs, and POAF.

To be specific, in order to confirm the differential expression, we first should collect blood samples from the patients and test the expression of the identified genes and circRNAs. The nomogram needs to be modified after the basic clinical characteristics have been adjusted. To investigate whether overexpression or downregulation of the related genes and circRNAs can decrease the incidence of POAF, basic research models should be developed. The precise mechanism can then be explored utilizing a number of omics techniques based on the confirmed results.

## Conclusion

In conclusion, our study identified four candidate diagnostic hub genes (*C1QA, C1R, MET, SDC4*) for diagnosing POAF compared with SR using integrated bioinformatics approaches and machine learning algorithms. The nomogram for diagnosis was constructed and the diagnostic value of each gene and the nomogram was ideal using ROC curve. The enrichment and immune cell infiltration analysis revealed that these genes were mainly enriched in immune regulation and that macrophages played the critical role in POAF pathogenesis. In addition, we discovered a new circ_0007738 with greater than fourfold down-regulation in POAF and created the ceRNA network for further exploration.

## Data availability statement

The original contributions presented in the study are included in the article/**Supplementary Material**. Further inquiries can be directed to the corresponding author.

## Author contributions

Hypothesis development: ZY. Study design, data acquisition and analysis: YZ, QW, GN. Image processing: YH, SX, CL. All the authors contributed to the article and approved the submitted version.

## Acknowledgment

We thank Dr. Liu (Nucleobase translocation of bioinformatics) for putting forward several valuable suggestions in the revision process.

## Conflict of interest

The authors declare that the research was conducted in the absence of any commercial or financial relationships that could be construed as a potential conflict of interest.

## Publisher’s note

All claims expressed in this article are solely those of the authors and do not necessarily represent those of their affiliated organizations, or those of the publisher, the editors and the reviewers. Any product that may be evaluated in this article, or claim that may be made by its manufacturer, is not guaranteed or endorsed by the publisher.
