# Supplementary material for: Immune-associated pivotal biomarkers identification and competing endogenous RNA network construction in post-operative atrial fibrillation by comprehensive bioinformatics and machine learning strategies
Source: Front Immunol. 2022 Oct 20;13:974935. doi: 10.3389/fimmu.2022.974935 (PMC9630466; doi:10.3389/fimmu.2022.974935)
Supplement: Supplementary file 1 [file Table_1.docx]

**Supplementary Material**

**Supplementary Table S1.** Complete list of DEGs regarding POAF compared with SR via GSE143924.

| Gene | logFC | P.Value | adj.P.Val |
| --- | --- | --- | --- |
| HLA-DQA1 | 1.651402641 | 0.006305249 | 0.645690168 |
| MIR4759 | 1.432072916 | 0.01190239 | 0.645690168 |
| TRAJ14 | 1.282750563 | 0.02581691 | 0.661668008 |
| TRAJ21 | 1.279189528 | 0.037737226 | 0.661668008 |
| TRAJ31 | 1.249807668 | 0.036024396 | 0.661668008 |
| MIR143 | 1.164013174 | 0.003934918 | 0.632731174 |
| LOC105373686 | 1.123166908 | 0.002029228 | 0.632731174 |
| TRAJ27 | 1.087068357 | 0.048696252 | 0.670692671 |
| MIR555 | 1.078225108 | 0.001135183 | 0.632731174 |
| TRAJ5 | 1.054464055 | 0.042722659 | 0.661668008 |
| TRAJ13 | 1.025283019 | 0.048886532 | 0.670692671 |
| TRAJ28 | 0.999755818 | 0.025374991 | 0.661668008 |
| CD24 | 0.969762648 | 0.005240994 | 0.632731174 |
| FCER2 | 0.960105605 | 0.005424887 | 0.632731174 |
| LOC105375488 | 0.957843399 | 0.000222655 | 0.632731174 |
| TRAJ48 | 0.954197795 | 0.01095951 | 0.645690168 |
| TRAJ52 | 0.94574571 | 0.039783605 | 0.661668008 |
| TRAJ7 | 0.942369731 | 0.040874831 | 0.661668008 |
| LEF1 | 0.934150786 | 0.04396235 | 0.661668008 |
| TMEM156 | 0.925711684 | 0.043515381 | 0.661668008 |
| TRAJ44 | 0.910960757 | 0.014188166 | 0.645690168 |
| TRAJ12 | 0.907998111 | 0.046611777 | 0.669331344 |
| LOC105377134 | 0.904583557 | 0.001753813 | 0.632731174 |
| CARMN | 0.894393757 | 0.005152035 | 0.632731174 |
| LOC284454 | 0.863865306 | 0.023311396 | 0.661668008 |
| CCL2 | 0.861013139 | 0.012331006 | 0.645690168 |
| IGKV1D-13 | 0.851746728 | 0.039673437 | 0.661668008 |
| CYSLTR1 | 0.83487714 | 0.008491788 | 0.645690168 |
| SNORD115-30 | 0.812650973 | 0.014315364 | 0.645690168 |
| MAB21L1 | 0.810414291 | 0.046984385 | 0.670692671 |
| COX14 | 0.807827496 | 0.003523331 | 0.632731174 |
| TRAJ53 | 0.798672503 | 0.041162732 | 0.661668008 |
| LOC105379251 | 0.798473832 | 0.011223412 | 0.645690168 |
| MIR604 | 0.793801217 | 0.03128331 | 0.661668008 |
| PPIAL4C | 0.78766721 | 0.015675844 | 0.654622156 |
| TRGC2 | 0.782049012 | 0.043844242 | 0.661668008 |
| C2CD4B | 0.758418626 | 0.000764311 | 0.632731174 |
| FCRL3 | 0.757448184 | 0.042878682 | 0.661668008 |
| TRAJ50 | 0.754255126 | 0.032972055 | 0.661668008 |
| TRAJ23 | 0.754092241 | 0.020513615 | 0.661668008 |
| IGLV2-8 | 0.752966777 | 0.007159717 | 0.645690168 |
| IFNG-AS1 | 0.742319135 | 0.039197421 | 0.661668008 |
| BLOC1S1-RDH5 | 0.739195682 | 0.021064345 | 0.661668008 |
| BLNK | 0.70984438 | 0.012469913 | 0.645690168 |
| ABCC6P2 | 0.705615988 | 0.007417691 | 0.645690168 |
| GUSBP4 | 0.696680445 | 0.007224223 | 0.645690168 |
| LOC101928067 | 0.696426971 | 0.003723374 | 0.632731174 |
| LOC105372607 | 0.696405459 | 0.048837907 | 0.670692671 |
| LINC00926 | 0.693504087 | 0.046758883 | 0.669331344 |
| MIR3137 | 0.690293707 | 0.003935532 | 0.632731174 |
| TRBV30 | 0.686728744 | 0.032330841 | 0.661668008 |
| HIST1H4A | 0.681071715 | 0.023158272 | 0.661668008 |
| SNORA70E | 0.679393744 | 0.009879796 | 0.645690168 |
| EDNRA | 0.676202758 | 0.025320047 | 0.661668008 |
| LOC105374360 | 0.66788475 | 0.005717312 | 0.632731174 |
| LOC105369652 | 0.665954674 | 0.027618064 | 0.661668008 |
| GPR15 | 0.66458442 | 0.013712666 | 0.645690168 |
| LOC101060254 | 0.661990078 | 0.000249769 | 0.632731174 |
| MIR6748 | 0.660197472 | 0.009264108 | 0.645690168 |
| MIR27A | 0.656582867 | 0.046274023 | 0.668028502 |
| PLK2 | 0.652662037 | 0.006121052 | 0.645690168 |
| LINC01336 | 0.646570631 | 0.017142218 | 0.660463173 |
| LOC105379689 | 0.646053796 | 0.02183021 | 0.661668008 |
| MIR548X | 0.644951989 | 0.04128489 | 0.661668008 |
| TRAV5 | 0.635034343 | 0.045293153 | 0.666125982 |
| LOC100996770 | 0.633461676 | 0.015165557 | 0.645690168 |
| CXorf65 | 0.631611978 | 0.029659267 | 0.661668008 |
| LOC101929531 | 0.62871531 | 0.002839147 | 0.632731174 |
| LINC01480 | 0.626124271 | 0.007518299 | 0.645690168 |
| SPRR2E | 0.625962928 | 0.017445327 | 0.660463173 |
| MIR4421 | 0.623167582 | 0.010838999 | 0.645690168 |
| ANKRD20A5P | 0.622313869 | 0.022440598 | 0.661668008 |
| FAM159A | 0.618473188 | 0.016963468 | 0.660463173 |
| IER2 | 0.610187931 | 0.030857628 | 0.661668008 |
| PRSS3P2 | 0.609179216 | 0.035805444 | 0.661668008 |
| DGAT2 | 0.607081368 | 0.029939064 | 0.661668008 |
| LOC105379555 | 0.605466368 | 0.041249584 | 0.661668008 |
| LOC105378171 | 0.604069359 | 0.008754108 | 0.645690168 |
| ADRA2A | 0.599915945 | 0.03474456 | 0.661668008 |
| CAMK4 | 0.598910963 | 0.046182024 | 0.668028502 |
| DEF6 | 0.587383805 | 0.022905655 | 0.661668008 |
| GLCCI1 | 0.586158662 | 0.002761279 | 0.632731174 |
| LOC105371815 | -0.58621125 | 0.013596669 | 0.645690168 |
| BICC1 | -0.586663212 | 0.027750882 | 0.661668008 |
| LOC440149 | -0.588631107 | 0.006250986 | 0.645690168 |
| GPX8 | -0.589348892 | 0.002424543 | 0.632731174 |
| LOC102724210 | -0.591183344 | 0.049278877 | 0.670692671 |
| SDC4 | -0.591384147 | 0.004541015 | 0.632731174 |
| CYP27A1 | -0.592189318 | 0.034049978 | 0.661668008 |
| PAMR1 | -0.593279853 | 0.032485926 | 0.661668008 |
| C1QA | -0.593425323 | 0.012201259 | 0.645690168 |
| TPRG1-AS2 | -0.596891352 | 0.015067819 | 0.645690168 |
| LOC105373850 | -0.598101272 | 0.010198924 | 0.645690168 |
| MLIP | -0.599711033 | 0.010661816 | 0.645690168 |
| ARFGEF3 | -0.600534326 | 0.020315975 | 0.661668008 |
| LOC105378380 | -0.601336205 | 0.043504434 | 0.661668008 |
| COL12A1 | -0.601567785 | 0.031891889 | 0.661668008 |
| SGPP2 | -0.60179729 | 0.035327214 | 0.661668008 |
| USP53 | -0.602992633 | 0.033448403 | 0.661668008 |
| TNXB | -0.605987876 | 0.038165624 | 0.661668008 |
| TLR2 | -0.607560328 | 0.025166203 | 0.661668008 |
| C1R | -0.609406936 | 0.001958509 | 0.632731174 |
| VTN | -0.609845647 | 0.009170196 | 0.645690168 |
| C10orf113 | -0.611701401 | 0.033476331 | 0.661668008 |
| SLC19A2 | -0.61260062 | 0.030781115 | 0.661668008 |
| HAS2 | -0.613211986 | 0.035128829 | 0.661668008 |
| MFI2 | -0.613653359 | 0.003198482 | 0.632731174 |
| GRID2 | -0.613941306 | 0.043477123 | 0.661668008 |
| MIR450B | -0.614449168 | 0.017754728 | 0.661668008 |
| LOC648570 | -0.61540405 | 0.033624599 | 0.661668008 |
| AMOT | -0.616121174 | 0.005542727 | 0.632731174 |
| TNXA | -0.619823294 | 0.034370824 | 0.661668008 |
| FOSL2 | -0.6201892 | 0.039559859 | 0.661668008 |
| LOC102723973 | -0.621360585 | 0.038037642 | 0.661668008 |
| KLF15 | -0.62281748 | 0.027442436 | 0.661668008 |
| MET | -0.624324758 | 0.007136098 | 0.645690168 |
| COL8A1 | -0.6249127 | 0.013252886 | 0.645690168 |
| ATP8A2 | -0.624917523 | 0.017084584 | 0.660463173 |
| TPD52L1 | -0.625745644 | 0.001720132 | 0.632731174 |
| CPM | -0.626762201 | 0.034592272 | 0.661668008 |
| LOC441728 | -0.629482875 | 0.010002231 | 0.645690168 |
| KLK11 | -0.629764316 | 0.042199392 | 0.661668008 |
| NEBL | -0.632918866 | 0.02576908 | 0.661668008 |
| DAPK1 | -0.63354036 | 0.034103376 | 0.661668008 |
| ADAMTS19 | -0.633733544 | 0.003774054 | 0.632731174 |
| VTRNA1-2 | -0.634693319 | 0.023846933 | 0.661668008 |
| LOC100287290 | -0.638807522 | 0.024867332 | 0.661668008 |
| SOHLH2 | -0.639050461 | 0.020376136 | 0.661668008 |
| ANO5 | -0.639986814 | 0.009524368 | 0.645690168 |
| LOC101928605 | -0.642139371 | 0.021878598 | 0.661668008 |
| CADM3 | -0.642442208 | 0.022535751 | 0.661668008 |
| MGAT4C | -0.643031386 | 0.012231929 | 0.645690168 |
| SNORD114-9 | -0.647845991 | 0.009499802 | 0.645690168 |
| HHIPL2 | -0.650556303 | 0.002462828 | 0.632731174 |
| LOC646762 | -0.654690988 | 0.033188852 | 0.661668008 |
| KLHL4 | -0.655423511 | 0.017391178 | 0.660463173 |
| LOC105375796 | -0.655735731 | 0.005589312 | 0.632731174 |
| MRC2 | -0.656734386 | 0.004503706 | 0.632731174 |
| KLF9 | -0.661853051 | 0.034503451 | 0.661668008 |
| SMPD3 | -0.662287194 | 0.048987925 | 0.670692671 |
| PDPN | -0.667496326 | 0.02004166 | 0.661668008 |
| LOC105377726 | -0.667653543 | 0.04964734 | 0.670692671 |
| ADGRG6 | -0.675955936 | 0.044851397 | 0.666125982 |
| LOC105377460 | -0.676819543 | 0.026004354 | 0.661668008 |
| CHI3L1 | -0.682429955 | 0.024420327 | 0.661668008 |
| LOC105374728 | -0.684851215 | 0.002410979 | 0.632731174 |
| ZNF812P | -0.689190658 | 0.04002835 | 0.661668008 |
| MIR622 | -0.690756729 | 0.020841913 | 0.661668008 |
| MS4A6A | -0.691880971 | 0.039110603 | 0.661668008 |
| SNORD114-1 | -0.695244976 | 0.028251291 | 0.661668008 |
| GAS7 | -0.698765502 | 0.022876054 | 0.661668008 |
| LINC01284 | -0.703226335 | 0.000791059 | 0.632731174 |
| SCARA5 | -0.70604557 | 0.042597214 | 0.661668008 |
| LTBP1 | -0.707231599 | 0.024367822 | 0.661668008 |
| WWC1 | -0.708205182 | 0.022014506 | 0.661668008 |
| MIR548V | -0.710434889 | 0.009670013 | 0.645690168 |
| IRAK3 | -0.71051868 | 0.003330084 | 0.632731174 |
| LOC100129473 | -0.716344806 | 0.005085017 | 0.632731174 |
| MIR4632 | -0.717551705 | 0.014184875 | 0.645690168 |
| FIBIN | -0.717694501 | 0.041938596 | 0.661668008 |
| HPSE | -0.71922937 | 0.020703782 | 0.661668008 |
| LOC105374084 | -0.729805459 | 0.046808022 | 0.669331344 |
| MIR511 | -0.735092274 | 0.012678082 | 0.645690168 |
| C2 | -0.736371961 | 0.00714929 | 0.645690168 |
| C6 | -0.737752231 | 0.041446535 | 0.661668008 |
| MUM1L1 | -0.73889825 | 0.040763952 | 0.661668008 |
| SLC4A4 | -0.74307829 | 0.029598523 | 0.661668008 |
| MT1M | -0.747733474 | 0.028144891 | 0.661668008 |
| LRRC1 | -0.748475311 | 0.010820144 | 0.645690168 |
| GAS1 | -0.75557953 | 0.011746467 | 0.645690168 |
| HERC2P2 | -0.757041175 | 0.001953969 | 0.632731174 |
| PLLP | -0.758653352 | 0.024313466 | 0.661668008 |
| SNORD123 | -0.765183088 | 0.00197105 | 0.632731174 |
| PRR15 | -0.782317802 | 0.011796263 | 0.645690168 |
| HTRA3 | -0.783833845 | 0.030012838 | 0.661668008 |
| SEMA3B | -0.785538782 | 0.022581821 | 0.661668008 |
| LOC105371120 | -0.788394877 | 0.000136409 | 0.632731174 |
| CYP4B1 | -0.794992497 | 0.019617621 | 0.661668008 |
| LOC105377621 | -0.802643297 | 0.034200405 | 0.661668008 |
| TMEM56-RWDD3 | -0.80599972 | 0.018017035 | 0.661668008 |
| ADAMTS15 | -0.806397344 | 0.01069012 | 0.645690168 |
| LOC105379513 | -0.81067001 | 0.000415336 | 0.632731174 |
| BCO2 | -0.810888246 | 0.020768424 | 0.661668008 |
| LOC103171574 | -0.812293517 | 0.031292275 | 0.661668008 |
| MIR520E | -0.814433886 | 0.019356997 | 0.661668008 |
| FGFBP2 | -0.82194787 | 0.013884109 | 0.645690168 |
| LOC105373013 | -0.822868312 | 0.005379498 | 0.632731174 |
| PTN | -0.823020936 | 0.009833357 | 0.645690168 |
| MT1A | -0.825167941 | 0.048616692 | 0.670692671 |
| COL28A1 | -0.827510046 | 0.010924641 | 0.645690168 |
| SENP3-EIF4A1 | -0.827837972 | 0.006029395 | 0.645690168 |
| AOX1 | -0.828408312 | 0.032503939 | 0.661668008 |
| CLEC4E | -0.834463906 | 0.012762071 | 0.645690168 |
| OMD | -0.845334199 | 0.022443279 | 0.661668008 |
| LOC100507639 | -0.868586057 | 0.002491073 | 0.632731174 |
| GFPT2 | -0.871635632 | 0.01693948 | 0.660463173 |
| SNORA58 | -0.882367322 | 0.000494086 | 0.632731174 |
| PKP2 | -0.883491772 | 0.010424605 | 0.645690168 |
| LSAMP | -0.909645541 | 0.010477849 | 0.645690168 |
| FRAS1 | -0.915923952 | 0.041673313 | 0.661668008 |
| SULF1 | -0.935617482 | 0.024843594 | 0.661668008 |
| MUC16 | -0.938931951 | 0.043792429 | 0.661668008 |
| ADGRD1 | -0.944407897 | 0.005281015 | 0.632731174 |
| MS4A4A | -0.954980373 | 0.022654758 | 0.661668008 |
| RARRES1 | -0.98509411 | 0.003919438 | 0.632731174 |
| MT1E | -0.991485701 | 0.02207031 | 0.661668008 |
| NNMT | -1.002746619 | 0.003299893 | 0.632731174 |
| BMP2 | -1.026236786 | 0.000501336 | 0.632731174 |
| MERTK | -1.034292415 | 0.008444409 | 0.645690168 |
| SLC39A8 | -1.054931046 | 0.04248748 | 0.661668008 |
| SERTM1 | -1.055580798 | 0.042469623 | 0.661668008 |
| CFB | -1.074406578 | 0.037083632 | 0.661668008 |
| FLRT3 | -1.074495434 | 0.02376634 | 0.661668008 |
| KCNT2 | -1.086010084 | 0.013703352 | 0.645690168 |
| SLED1 | -1.088067327 | 0.002756759 | 0.632731174 |
| SLC28A3 | -1.153258086 | 0.010325537 | 0.645690168 |
| PXDNL | -1.168406643 | 0.02071443 | 0.661668008 |
| FNDC1 | -1.188589009 | 0.009827006 | 0.645690168 |
| PER1 | -1.196820217 | 0.01208055 | 0.645690168 |
| SNORD115-46 | -1.220933109 | 0.005117069 | 0.632731174 |
| SLPI | -1.241233311 | 0.036119887 | 0.661668008 |
| TBX20 | -1.273664032 | 0.015691432 | 0.654622156 |
| CCL18 | -1.297693674 | 0.042322473 | 0.661668008 |
| LOC105375032 | -1.299383738 | 0.011225814 | 0.645690168 |
| MT1JP | -1.345125083 | 0.002220383 | 0.632731174 |
| ZBTB16 | -1.366638824 | 0.004292233 | 0.632731174 |
| LOC101926917 | -1.378831951 | 0.001926247 | 0.632731174 |
| PLA2G2A | -1.500013993 | 0.004817073 | 0.632731174 |
| CDH19 | -1.570635851 | 0.010376926 | 0.645690168 |
| ANXA8 | -1.599281649 | 0.029628023 | 0.661668008 |
| HP | -1.66727667 | 0.020789075 | 0.661668008 |
| CTSE | -1.709409573 | 0.008121116 | 0.645690168 |
| UPK3B | -1.78331507 | 0.016496302 | 0.660463173 |
| SYT4 | -1.95432629 | 0.030036872 | 0.661668008 |

Abbreviations: DEGs, differentially expressed genes; POAF, post-operative atrial fibrillation; SR, sinus rhythm; FC, fold change.

**Supplementary Table S2.** Complete list and the intersection of common genes from three algorithms via CytoHubba plug-in.

| Algorithms | | | Intersection | Annotation |
| --- | --- | --- | --- | --- |
| Betweenness | Closeness | Degree |  |  |
| VTN | VTN | VTN | VTN | Vitronectin |
| CCL2 | CFB | C1QA | CCL2 | C-C Motif Chemokine Ligand 2 |
| MET | C1R | CFB | MET | MET Proto-Oncogene, Receptor Tyrosine Kinase |
| C1QA | CCL2 | C1R | C1QA | Complement C1q A Chain |
| SDC4 | C1QA | CCL2 | SDC4 | Syndecan 4 |
| CFB | HP | TLR2 | CFB | Complement Factor B |
| C1R | C2 | C2 | C1R | Complement C1r |
| C6 | TLR2 | C6 | C6 | Complement C6 |
| HP | C6 | SDC4 | HP | Haptoglobin |
| ZBTB16 | SDC4 | MET | MFI2 | Melanotransferrin |
| BMP2 | MET | HP | COL8A1 | Collagen Type VIII Alpha 1 Chain |
| MFI2 | CCL18 | ZBTB16 | SCARA5 | Scavenger Receptor Class A Member 5 |
| COL8A1 | SCARA5 | MS4A4A | TLR2 | Toll receptor 2 |
| SCARA5 | MFI2 | CCL18 | C2 | Complement C2 |
| PAMR1 | COL8A1 | COL8A1 | MS4A4A | Membrane Spanning 4-Domains A4A |
| CD24 | MS4A4A | MFI2 |  |  |
| TLR2 | MS4A6A | MT1E |  |  |
| C2 | BMP2 | MS4A6A |  |  |
| LEF1 | CHI3L1 | SCARA5 |  |  |
| MS4A4A | CLEC4E | CHI3L1 |  |  |

**Supplementary Table S3.** Functional enrichment analysis regarding POAF compared with SR.

| ID | Description | Count | p.adjust | geneID |
| --- | --- | --- | --- | --- |
| **Biological Process** |  |  |  |  |
| GO:0042221 | response to chemical | 33 | 0.00133299 | IER2/MT1E/SLPI/GFPT2/NNMT/AMOT/MET/SULF1/  CCL18/TLR2/C2/HP/MT1M/C1QA/LTBP1/CHI3L1/  HLA-DQA1/MT1A/VTN/HAS2/PTN/ADRA2A/CCL2/  CYP4B1/BMP2/IRAK3/SMPD3/FCER2/LEF1/DAPK1/  CD24/FLRT3/SEMA3B |
| GO:0006950 | response to stress | 32 | 0.000305776 | C2CD4B/SLPI/MERTK/MET/C1R/C6/TLR2/C2/HPSE/  MUC16/HP/SCARA5/C1QA/CHI3L1/ANXA8/CLEC4E/  HLA-DQA1/VTN/HAS2/PTN/ADRA2A/CCL2/CFB/  BMP2/IRAK3/SMPD3/PLLP/PLA2G2A/DAPK1/CD24/  FLRT3/SDC4 |
| GO:0048731 | system development | 30 | 0.012875758 | IER2/ZBTB16/NNMT/GCNT4/NEBL/MERTK/AMOT/  MET/SULF1/C6/TLR2/HPSE/COL8A1/C1QA/LTBP1/  CHI3L1/CLEC4E/VTN/HAS2/PTN/CCL2/BMP2/IRAK3/  SMPD3/LEF1/CD24/FLRT3/MAB21L1/SDC4/SEMA3B |
| GO:0048583 | regulation of response to stimulus | 29 | 0.00802662 | C2CD4B/AMOT/MET/C1R/SULF1/CCL18/C6/TLR2/C2/  HPSE/MUC16/WWC1/C1QA/LTBP1/CHI3L1/CLEC4E/  HLA-DQA1/VTN/PTN/ADRA2A/CCL2/CFB/BMP2/  IRAK3/FCER2/PLA2G2A/LEF1/DAPK1/CD24 |
| GO:0002376 | immune system process | 28 | 0.000315014 | SLPI/ZBTB16/CTSE/MERTK/C1R/CCL18/C6/TLR2/C2/  HPSE/MUC16/HP/C1QA/CHI3L1/CLEC4E/HLA-DQA1/  VTN/PTN/CCL2/CFB/IRAK3/SMPD3/FCER2/PLA2G2A/  LEF1/DAPK1/CD24/SDC4 |
| GO:0070887 | cellular response to chemical stimulus | 25 | 0.002779967 | MT1E/GFPT2/MET/SULF1/TLR2/HP/MT1M/LTBP1/  CHI3L1/HLA-DQA1/MT1A/VTN/HAS2/PTN/ADRA2A/  CCL2/BMP2/IRAK3/SMPD3/FCER2/LEF1/DAPK1/  CD24/FLRT3/SEMA3B |
| GO:0030154 | cell differentiation | 25 | 0.03711531 | IER2/ZBTB16/NEBL/MERTK/AMOT/MET/SULF1/  TLR2/COL8A1/C1QA/SOHLH2/CLEC4E/VTN/HAS2/  PTN/CCL2/BMP2/SMPD3/PLA2G2A/LEF1/CD24/FLRT3/  COL12A1/SDC4/SEMA3B |
| GO:0009605 | response to external stimulus | 25 | 0.000244099 | C2CD4B/SLPI/AMOT/MET/C1R/CCL18/C6/TLR2/C2/  HPSE/HP/C1QA/CHI3L1/CLEC4E/VTN/PTN/CCL2/CFB/  BMP2/IRAK3/PLA2G2A/LEF1/CD24/FLRT3/SEMA3B |
| GO:0048513 | animal organ development | 24 | 0.015615903 | ZBTB16/NNMT/GCNT4/NEBL/MERTK/MET/SULF1/  TLR2/HPSE/COL8A1/LTBP1/CHI3L1/CLEC4E/VTN/  HAS2/PTN/CCL2/BMP2/SMPD3/LEF1/CD24/FLRT3/  MAB21L1/SDC4 |
| GO:0009653 | anatomical structure morphogenesis | 24 | 0.000725385 | ZBTB16/GCNT4/NEBL/MERTK/AMOT/MET/SULF1/  C6/TLR2/HPSE/COL8A1/CHI3L1/VTN/HAS2/PTN/CCL2/  BMP2/SMPD3/LEF1/FLRT3/MAB21L1/COL12A1/SDC4/  SEMA3B |
| **Cellular component** |  |  |  |  |
| GO:0005576 | extracellular region | 34 | 4.77055E-05 | SLPI/TNXB/PAMR1/ADAMTS19/NEBL/MERTK/MET/  C1R/AOX1/SULF1/C6/COL28A1/C2/ADAMTS15/HPSE/  MUC16/HP/COL8A1/C1QA/LTBP1/CHI3L1/ANXA8/  VTN/PTN/CCL2/CFB/KLK11/BMP2/FCER2/PLA2G2A/  FLRT3/COL12A1/SDC4/SEMA3B |
| GO:0044421 | extracellular region part | 29 | 5.41938E-05 | SLPI/TNXB/NEBL/MERTK/C1R/AOX1/SULF1/C6/  COL28A1/C2/ADAMTS15/MUC16/HP/COL8A1/C1QA/  LTBP1/CHI3L1/ANXA8/VTN/PTN/CFB/KLK11/BMP2/  FCER2/PLA2G2A/FLRT3/COL12A1/SDC4/SEMA3B |
| GO:0012505 | endomembrane system | 25 | 0.046806483 | SLPI/GCNT4/CTSE/SULF1/TLR2/COL28A1/HPSE/  MUC16/HP/COL8A1/LTBP1/CHI3L1/ANXA8/  HLA-DQA1/VTN/PTN/CYP4B1/KLK11/SMPD3/SGPP2/  PLA2G2A/FLRT3/COL12A1/SDC4/SEMA3B |
| GO:0005615 | extracellular space | 24 | 0.002975737 | SLPI/TNXB/NEBL/MERTK/C1R/AOX1/SULF1/C6/C2/  ADAMTS15/MUC16/HP/C1QA/CHI3L1/VTN/PTN/CFB/  KLK11/BMP2/FCER2/PLA2G2A/FLRT3/COL12A1/SDC4 |
| GO:0031982 | vesicle | 23 | 0.046806483 | SLPI/TNXB/NEBL/CTSE/AMOT/C1R/AOX1/C6/TLR2/  C2/HPSE/MUC16/HP/CHI3L1/ANXA8/HLA-DQA1/  VTN/CFB/KLK11/FCER2/PLA2G2A/COL12A1/SDC4 |
| GO:0070062 | extracellular exosome | 17 | 0.01990889 | SLPI/TNXB/NEBL/C1R/AOX1/C6/C2/MUC16/HP/  CHI3L1/VTN/CFB/KLK11/FCER2/PLA2G2A/COL12A1/  SDC4 |
| GO:1903561 | extracellular vesicle | 17 | 0.01990889 | SLPI/TNXB/NEBL/C1R/AOX1/C6/C2/MUC16/HP/  CHI3L1/VTN/CFB/KLK11/FCER2/PLA2G2A/COL12A1/  SDC4 |
| GO:0043230 | extracellular organelle | 17 | 0.01990889 | SLPI/TNXB/NEBL/C1R/AOX1/C6/C2/MUC16/HP/  CHI3L1/VTN/CFB/KLK11/FCER2/PLA2G2A/COL12A1/  SDC4 |
| GO:0031012 | extracellular matrix | 15 | 1.74056E-08 | SLPI/TNXB/SULF1/COL28A1/ADAMTS15/COL8A1/  C1QA/LTBP1/CHI3L1/ANXA8/VTN/PTN/FLRT3/  COL12A1/SEMA3B |
| GO:0005783 | endoplasmic reticulum | 14 | 0.04390735 | SULF1/COL28A1/COL8A1/LTBP1/CHI3L1/HLA-DQA1/  VTN/PTN/CYP4B1/SGPP2/PLA2G2A/FLRT3/COL12A1/  SEMA3B |
| **Molecular function** |  |  |  |  |
| GO:0005201 | extracellular matrix structural constituent | 7 | 0.000733743 | TNXB/COL28A1/COL8A1/LTBP1/CHI3L1/VTN/  COL12A1 |
| GO:0004175 | endopeptidase activity | 7 | 0.028947563 | ADAMTS19/CTSE/C1R/C2/ADAMTS15/  CFB/KLK11 |
| GO:0005539 | glycosaminoglycan binding | 5 | 0.04240358 | TNXB/TLR2/ADAMTS15/VTN/PTN |
| GO:0005178 | integrin binding | 4 | 0.04240358 | TNXB/VTN/PTN/FCER2 |
| GO:0030020 | extracellular matrix structural constituent conferring tensile strength | 3 | 0.036938646 | COL28A1/COL8A1/COL12A1 |
| GO:0045545 | syndecan binding | 2 | 0.028947563 | HPSE/PTN |
| GO:0045499 | chemorepellent activity | 2 | 0.036938646 | FLRT3/SEMA3B |
| **KEGG pathway** |  |  |  |  |
| hsa04610 | Complement and coagulation cascades | 6 | 0.000328008 | C1R/C6/C2/C1QA/VTN/CFB |
| hsa05150 | Staphylococcus aureus infection | 5 | 0.007402215 | C1R/C2/C1QA/HLA-DQA1/CFB |
| hsa05322 | Systemic lupus erythematosus | 5 | 0.022546995 | C1R/C6/C2/C1QA/HLA-DQA1 |
| hsa05205 | Proteoglycans in cancer | 5 | 0.075086671 | MET/TLR2/HPSE/VTN/SDC4 |
| hsa04390 | Hippo signaling pathway | 4 | 0.117741014 | AMOT/WWC1/BMP2/LEF1 |
| hsa05144 | Malaria | 3 | 0.05946 | MET/TLR2/CCL2 |
| hsa04978 | Mineral absorption | 3 | 0.075086671 | MT1E/MT1M/MT1A |
| hsa05133 | Pertussis | 3 | 0.117709252 | C1R/C2/C1QA |
| hsa04512 | ECM-receptor interaction | 3 | 0.131092158 | TNXB/VTN/SDC4 |
| hsa05323 | Rheumatoid arthritis | 3 | 0.131092158 | TLR2/HLA-DQA1/CCL2 |

Abbreviations: POAF, post-operative atrial fibrillation; SR, sinus rhythm; KEGG, Kyoto Encyclopedia of Genes and Genomes.

**Supplementary Table S4.** Complete list of DECs regarding POAF compared with SR via GSE97455.

| ID | logFC | P.Value | adj.P.Val |
| --- | --- | --- | --- |
| hsa_circ_0037798 | -1.317766 | 4.35E-09 | 1.45E-05 |
| hsa_circ_0000367 | 3.4703945 | 8.85E-08 | 0.0001268 |
| hsa_circ_0055387 | 1.0480251 | 2.66E-07 | 0.000222 |
| hsa_circ_0091000 | 1.1065588 | 5.55E-07 | 0.0003423 |
| hsa_circ_0056558 | 1.1308336 | 1.73E-06 | 0.0005983 |
| hsa_circ_0005899 | 1.1572911 | 3.83E-06 | 0.0009221 |
| hsa_circ_0005571 | 1.1348375 | 5.05E-06 | 0.0011244 |
| hsa_circ_0001666 | 2.11165 | 5.20E-06 | 0.0011347 |
| hsa_circ_0005568 | 1.0258542 | 6.09E-06 | 0.0011885 |
| hsa_circ_0089974 | -1.318669 | 7.03E-06 | 0.0013306 |
| hsa_circ_0000288 | 2.1956524 | 8.00E-06 | 0.0014341 |
| hsa_circ_0092283 | -1.241269 | 8.09E-06 | 0.0014341 |
| hsa_circ_0021647 | 1.8532526 | 1.13E-05 | 0.0018897 |
| hsa_circ_0021652 | 2.0148074 | 1.18E-05 | 0.0019392 |
| hsa_circ_0078346 | -1.096086 | 1.36E-05 | 0.0021322 |
| hsa_circ_0088036 | -1.217361 | 2.08E-05 | 0.0027873 |
| hsa_circ_0006349 | -1.144248 | 2.22E-05 | 0.0028566 |
| hsa_circ_0065649 | 1.0556799 | 2.44E-05 | 0.0030631 |
| hsa_circ_0043278 | 1.9888882 | 2.80E-05 | 0.0034216 |
| hsa_circ_0077007 | 1.2466567 | 5.43E-05 | 0.005285 |
| hsa_circ_0007060 | -1.139265 | 6.84E-05 | 0.0061267 |
| hsa_circ_0071312 | -1.008539 | 0.000100807 | 0.0075447 |
| hsa_circ_0047771 | -1.031846 | 0.000109952 | 0.0077182 |
| hsa_circ_0000691 | 2.4159217 | 0.000130508 | 0.0087153 |
| hsa_circ_0002701 | 1.0082757 | 0.0002506 | 0.0128228 |
| hsa_circ_0010884 | 1.2714131 | 0.000310906 | 0.0147776 |
| hsa_circ_0017854 | -1.053622 | 0.000316541 | 0.0148799 |
| hsa_circ_0007874 | 1.2803663 | 0.000341757 | 0.0156293 |
| hsa_circ_0036567 | 1.2446784 | 0.000362434 | 0.0160126 |
| hsa_circ_0030162 | 2.0127084 | 0.000365101 | 0.0160596 |
| hsa_circ_0051218 | 1.2278528 | 0.000569911 | 0.0212477 |
| hsa_circ_0050649 | -1.169171 | 0.000644627 | 0.022526 |
| hsa_circ_0000320 | 1.0872015 | 0.000651338 | 0.022603 |
| hsa_circ_0006168 | 2.1548125 | 0.000680231 | 0.0232039 |
| hsa_circ_0006220 | 1.4553769 | 0.000762662 | 0.024359 |
| hsa_circ_0082317 | -1.051227 | 0.000805254 | 0.0251585 |
| hsa_circ_0000680 | -1.123189 | 0.000829745 | 0.0256837 |
| hsa_circ_0018722 | 1.2495163 | 0.000838813 | 0.0258657 |
| hsa_circ_0000788 | 1.0307203 | 0.000908022 | 0.027124 |
| hsa_circ_0080638 | -1.160988 | 0.001217664 | 0.0332751 |
| hsa_circ_0000326 | 1.686739 | 0.001254235 | 0.0338734 |
| hsa_circ_0060144 | -1.00519 | 0.00137482 | 0.0358858 |
| hsa_circ_0001605 | 1.0100226 | 0.001376941 | 0.0358858 |
| hsa_circ_0014352 | 1.3824827 | 0.001444734 | 0.0369623 |
| hsa_circ_0007503 | -1.007769 | 0.001733675 | 0.040929 |
| hsa_circ_0078264 | -1.474057 | 0.002026462 | 0.0449607 |
| hsa_circ_0000222 | 1.14069 | 0.002746775 | 0.0544415 |
| hsa_circ_0050648 | -1.067708 | 0.003196441 | 0.0594353 |
| hsa_circ_0025016 | 2.0280111 | 0.003290927 | 0.0604482 |
| hsa_circ_0002082 | 1.8975749 | 0.003587245 | 0.0635606 |
| hsa_circ_0063331 | 1.2220665 | 0.003632111 | 0.0640183 |
| hsa_circ_0000812 | -1.059478 | 0.004054879 | 0.0680657 |
| hsa_circ_0004183 | 1.4451293 | 0.006950239 | 0.0926914 |
| hsa_circ_0001955 | -1.012681 | 0.007611092 | 0.0962568 |
| hsa_circ_0000514 | 2.0614273 | 0.00776068 | 0.0972228 |
| hsa_circ_0007738 | -2.770254 | 0.007991238 | 0.0987256 |
| hsa_circ_0000515 | 1.8893432 | 0.008863059 | 0.1056543 |
| hsa_circ_0079385 | 1.0846679 | 0.009352454 | 0.1093191 |
| hsa_circ_0006853 | 2.1403583 | 0.010489011 | 0.1185956 |
| hsa_circ_0023216 | -1.038588 | 0.012018062 | 0.1276001 |
| hsa_circ_0000511 | 2.2961576 | 0.012394585 | 0.1300265 |
| hsa_circ_0000512 | 2.2387919 | 0.012720917 | 0.1313731 |
| hsa_circ_0001490 | 1.1490107 | 0.014095346 | 0.1388627 |
| hsa_circ_0071271 | -1.591208 | 0.023755132 | 0.1886489 |
| hsa_circ_0000325 | 1.0215695 | 0.02476324 | 0.192609 |
| hsa_circ_0006148 | -1.157974 | 0.02518465 | 0.1942586 |
| hsa_circ_0063329 | 1.4244482 | 0.033549847 | 0.2306178 |
| hsa_circ_0069977 | -1.275989 | 0.048066871 | 0.2788101 |

Abbreviations: DECs, differentially expressed circRNAs; POAF, post-operative atrial fibrillation; SR, sinus rhythm; FC, fold change.

**Supplementary Table S5.** MRNA-miRNA and circRNA-miRNA pairs for building ceRNA network.

| Node1 | Node2 |
| --- | --- |
| miRNA-circRNA pairs |  |
| hsa-miR-3928-5p | hsa_circ_0007738 |
| hsa-miR-3925-5p | hsa_circ_0007738 |
| hsa-miR-4476 | hsa_circ_0007738 |
| hsa-miR-6738-3p | hsa_circ_0007738 |
| hsa-miR-6876-5p | hsa_circ_0007738 |
| hsa-miR-6747-5p | hsa_circ_0007738 |
| hsa-miR-6812-5p | hsa_circ_0007738 |
| hsa-miR-6819-5p | hsa_circ_0007738 |
| miRNA-mRNA pairs |  |
| hsa-miR-3928-5p | C1QA |
| hsa-miR-3925-5p | SDC4 |
| hsa-miR-4476 | SDC4 |
| hsa-miR-6738-3p | SDC4 |
| hsa-miR-6876-5p | SDC4 |
| hsa-miR-6747-5p | MET |
| hsa-miR-6812-5p | MET |
| hsa-miR-6819-5p | MET |

Abbreviation: ceRNA, competing endogenous RNA.
